# Shared Genetic Signals of Hypoxia Adaptation in *Drosophila* and in High-Altitude Human Populations

**DOI:** 10.1093/molbev/msv248

**Published:** 2015-11-17

**Authors:** Aashish R. Jha, Dan Zhou, Christopher D. Brown, Martin Kreitman, Gabriel G. Haddad, Kevin P. White

**Affiliations:** ^1^Institute for Genomics and Systems Biology, The University of Chicago; ^2^Department of Human Genetics, The University of Chicago; ^3^Department of Ecology and Evolution, The University of Chicago; ^4^Division of Respiratory Medicine, Department of Pediatrics, University of California at San Diego; ^5^Committee on Genetics, Genomics and Systems Biology, The University of Chicago; ^6^Department of Neurosciences, University of California at San Diego; ^7^Rady Children’s Hospital, San Diego, CA

**Keywords:** experimental evolution, evolution, adaptation, hypoxia, high-altitude adaptation, evolve and resequence, pooled sequencing, polygenic traits, complex traits

## Abstract

The ability to withstand low oxygen (hypoxia tolerance) is a polygenic and mechanistically conserved trait that has important implications for both human health and evolution. However, little is known about the diversity of genetic mechanisms involved in hypoxia adaptation in evolving populations. We used experimental evolution and whole-genome sequencing in *Drosophila melanogaster* to investigate the role of natural variation in adaptation to hypoxia. Using a generalized linear mixed model we identified significant allele frequency differences between three independently evolved hypoxia-tolerant populations and normoxic control populations for approximately 3,800 single nucleotide polymorphisms. Around 50% of these variants are clustered in 66 distinct genomic regions. These regions contain genes that are differentially expressed between hypoxia-tolerant and normoxic populations and several of the differentially expressed genes are associated with metabolic processes. Additional genes associated with respiratory and open tracheal system development also show evidence of directional selection. RNAi-mediated knockdown of several candidate genes’ expression significantly enhanced survival in severe hypoxia. Using genomewide single nucleotide polymorphism data from four high-altitude human populations—Sherpas, Tibetans, Ethiopians, and Andeans, we found that several human orthologs of the genes under selection in flies are also likely under positive selection in all four high-altitude human populations. Thus, our results indicate that selection for hypoxia tolerance can act on standing genetic variation in similar genes and pathways present in organisms diverged by hundreds of millions of years.

## Introduction

The first appearance of eukaryotic cells 2 Ga and the subsequent evolution and diversification of multicellular organisms over the past 500 My have both been linked to the use of oxygen for metabolic energy (Knoll and Carroll 1999; Jiang et al. 2012). Hypoxic stress ensues when organisms are exposed to oxygen levels lower than those to which they are adapted (sea level = 21%). When populations of organisms migrate into new environments for multiple generations, evolutionary adaptation to hypoxia may occur. Given the heterogeneity of oxygen levels among the many different environments inhabited on Earth, it is not surprising that hypoxia adaptation has been a recurring theme in evolution, including in multiple human populations living at high altitudes in the Himalaya, the Andes, and the Ethiopian highlands.

Several positively selected genes have been documented in high-altitude Tibetans, Andeans, and Ethiopians (Wouters and Koritzinsky 2008; Bigham et al. 2009, 2010; Beall et al. 2010; Simonson et al. 2010; Yi et al. 2010; Alkorta-Aranburu et al. 2012; Scheinfeldt et al. 2012; Huerta-Sanchez et al. 2013; Zhou et al. 2013; Eichstaedt et al. 2014; Jeong et al. 2014; Udpa et al. 2014). However, it has been difficult to determine how many of these genes are involved in hypoxia adaptation for a number of reasons, including but not limited to heterogeneous environments at high altitudes and complicated demographic histories of high-altitude human populations. Hence, most studies have begun focusing on a handful of candidate genes in the HIF pathway, such as *EPAS1* (*HIF2α*) and *EGLN1* (*PHD2*) (Moore et al. 2004). However, previous studies have also shown that hypoxia tolerance is a polygenic trait and involves genes in HIF-independent pathways (Wouters and Koritzinsky 2008; Li et al. 2013). Many of these genes are likely to be under selection in high-altitude human populations (Alkorta-Aranburu et al. 2012; Huerta-Sanchez et al. 2013; Zhou et al. 2013; Eichstaedt et al. 2014; Jeong et al. 2014; Udpa et al. 2014). However, identifying which sets of genes are involved in hypoxia adaptation in high-altitude human populations still remains a challenge.

Experimental evolution followed by resequencing has identified genetic basis of many polygenic traits in *Drosophila melanogaster* (Burke et al. 2010; Turner et al. 2011; Zhou et al. 2011; Turner and Miller 2012; Orozco-terWengel et al. 2012; Remolina et al. 2012; Cassidy et al. 2013; Tobler et al. 2014; Jha et al. 2015). We reasoned that experimental evolution might be an excellent approach to characterize the genetic basis of hypoxia adaptation, particularly since *D. melanogaster* has been a very effective model system to study hypoxia tolerance (Liu et al. 2006; Zhou et al. 2007, 2008, 2011; Dekanty et al. 2010; Azad et al. 2012). *Drosophila* possesses a small genome with low levels of linkage disequilibrium, has relatively short generation time, large replicate populations can be derived from the same ancestral population, and can be easily maintained in controlled environmental conditions where hypoxia is the major selective force. In addition, genes responding to hypoxia in flies may also be relevant to humans because large numbers of genes—including those that are involved in oxygen sensing, metabolism, and respiratory system development—are evolutionarily conserved between flies and humans (Ghabrial et al. 2003). Comparison of hypoxia response between flies and humans may illuminate novel aspects of human genetic responses that are relevant to human high-altitude adaptation as well as in diseases.

Previously, we have used experimental evolution in *Drosophila* to generate populations that have adapted to severe hypoxia and were able to survive at 5% O_2_ (Zhou et al. 2007). At the 18th generation, approximately 3,000 genes were differentially expressed between the hypoxia adapted fly populations (AF populations) and the normoxic control fly populations (CF populations), suggesting strong adaptive response to hypoxia in these laboratory selected populations (Zhou et al. 2008). However, only a handful of selective sweeps were identified in the AF populations after they had been subjected to 4% oxygen for over 100 generations (Zhou et al. 2011). The abundance of differentially expressed genes during the initial selective response but the lack of positively selected genes after long-term selection may indicate differences between the genetic underpinnings of the early and the late adaptive responses to hypoxia. Indeed, one might expect that the initial response to selection would be restricted to standing natural variation whereas later adaptive responses might involve newly arising mutations. Alternatively, if adaptation from standing variation persists then selective sweeps could appear to be very rare even in the later generations (Przeworski et al. 2005). These questions have remained unresolved in this case, as the evolution of genomic architecture in response to selection and the mode of hypoxia adaptation remain to be identified in these populations.

To identify genetic variants underlying hypoxia tolerance, we performed whole-genome pooled-sequencing for all three replicate AF populations at an early generation before exposure to severe hypoxia (fourth generation) and at a later generation after adaptation to severe hypoxic environment (17th generation). We also sequenced the three replicate CF populations from the same two generations. Although haplotype-based tests designed to identify adaptively driven reduction in heterozygosity (e.g., iHS [Voight et al. 2006], XP-EHH [Sabeti et al. 2007]) cannot be applied to pooled-sequencing data, systematic and reproducible changes in allele frequencies are expected in response to selection if the replicate laboratory-selected populations follow convergent evolutionary paths. In line with these expectations, we detected several thousand naturally existing polymorphisms that show reproducible changes in allele frequencies in response to hypoxia between the three replicates of AF and CF populations. These variants also showed consistent and reproducible allele frequency changes between the pre- and postselection AF populations but not in CF populations. Many of these variants are clustered in distinct genomic regions throughout the *Drosophila* genome. Knocking down the expression of genes harboring these variants strikingly enhanced survival under hypoxic conditions, demonstrating that the candidate genes are functionally relevant to hypoxia tolerance in *Drosophila.* Further investigation in four high-altitude human populations (Sherpas, Tibetans, Ethiopians, and Andeans) using genomewide single nucleotide polymorphism (SNP) data revealed that orthologs for many of these candidate genes harbor several highly differentiated SNPs, indicating that these genes are likely under positive selection in all four human populations. We propose that the shared candidate genes that we have identified are likely to be fundamental hypoxia response genes whose functions have remained conserved across animal species.

## Results

### Selection for Hypoxia Tolerance and Resulting Phenotypes

**Fig. 1.**
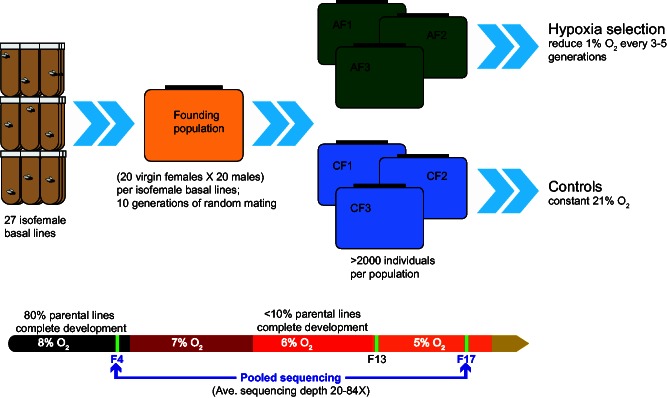
Schematic representation of experimental evolution. Top: Twenty-seven isofemale lines were used to create a founding population (yellow box), which was subdivided into six replicate subpopulations. Three of these subpopulations were maintained at room oxygen levels (21%) and were used as controls (CF, blue boxes) and three were exposed to hypoxia (AF, green boxes). Bottom: Low oxygen tolerance by AF populations. Hypoxia was initiated at 8% O_2_, which has little impact on development of parental lines. Although less than 10% of the parental lines yield any survivors at 6% O_2_, individuals from AF populations were able to survive and reproduce at even stronger hypoxic conditions (5% O_2_) by the 13th generation (F13). Blue arrows indicate the two generations (F4 and F17) that were sequenced.

A schematic representation of the experimental design is presented in figure 1. A freely mating base population, established from 27 isofemale lines collected at sea level, was divided into six replicate subpopulations. Three of these subpopulations were subjected to hypoxia by lowering the ambient oxygen concentration by 1% every 3–5 generations and three control populations were maintained in identical but normoxic conditions (21% O_2_). Although less than 10% of flies in the parental lines were able to complete development in hypoxia, the AF populations were able to complete development and propagate at 5% O_2_ by the 13th generation (Zhou et al. 2007). Also, hypoxia tolerance persisted even after exposing the AF populations to a normoxic environment for multiple generations (Zhou et al. 2007, 2008, 2011). These results indicate that an adaptive rather than physiological (acclimatization) response facilitated by the genetic variation for oxygen tolerance already present in the ancestral lines is likely responsible for hypoxia tolerance in the AF populations. In addition to adapting and tolerating very low oxygen environment, the AF populations displayed additional phenotypes, such as reduction in body weight, body size, cell number, and cell size (Zhou et al. 2007, 2008). These phenotypes have also been described in independent populations of *D. melanogaster* exposed to hypoxia (Peck and Maddrell 2005) and are consistent with previously established relationship between oxygen concentration, metabolism, and body size (Haldane 1926; Speakman 2005).

### Sequencing Statistics and Polymorphisms

We performed whole-genome sequencing using pooled genomic DNA from 100 flies from each of the replicate AF populations, using an early generation prior to severe hypoxia exposure (fourth generation, 8% O_2_) and a later generation where the flies had adapted to a normally lethal hypoxic condition (17th generation, 5% O_2_). Pooled DNA was also extracted and sequenced from 100 flies in CF populations from the 4th and 17th generations. We implemented a series of stringent filters (see Materials and Methods) to identify 1,075,949 bi-allelic positions relative to the *D. melanogaster* reference genome (supplementary fig. S1*A*, Supplementary Material online). More than 80% of all the variants identified in our study are also present in a collection of 163 lines of *D. melanogaster* from North Carolina that comprise the *D. melanogaster* Genetic Reference Panel (DGRP) sequences (Mackay et al. 2012), indicating that majority of the polymorphisms in our study are common polymorphisms widespread among natural *Drosophila* populations (supplementary fig. S1*B*, Supplementary Material online).

### Allele Frequency Divergence due to Hypoxia

**Fig. 2.**
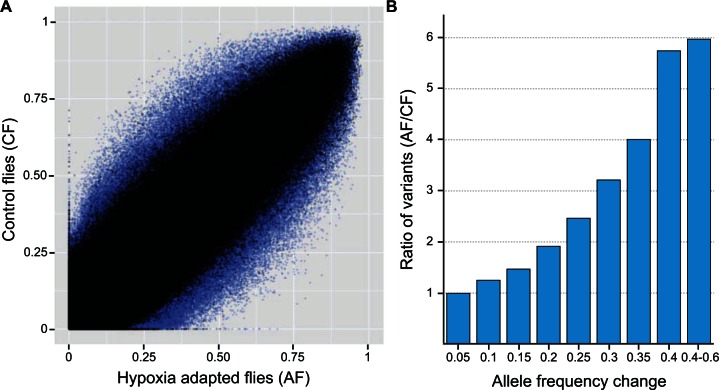
Allele frequency changes after hypoxia selection. (*A*) Allele frequencies in hypoxia adapted and normoxic control flies in the 4th generation (black) and in the 17th generation (blue). Each dot is the average allele frequency for each variant across the three replicates for each treatment. (*B*) Allele frequency changes between the 4th and 17th generations in AF populations relative to allele frequency changes between the 4th and 17th generations in CF populations. The *x* axis represents magnitude of allele frequency differences (in 5% bins). The *y* axis shows the ratio of number of variants that changed in frequencies represented by the values on the *x* axis. Ratios were calculated by counting the number of variants that changed at a given frequency in all three AF populations divided by the number of variants that changed at a given frequency in all three CF populations.

To evaluate whether there are genomic signatures of hypoxia selection, we first took a graphical approach to compare allele frequency changes in the AF and CF populations during selection. Although limited allele frequency differences were observed between the CF and AF populations at the fourth generation (fig. 2*A*, black), larger allele frequency changes were evident after 17 generations of hypoxia selection (fig. 2*A*, blue). To evaluate whether these allele frequency differences are specific to hypoxia adaptation, we calculated changes in allele frequencies between the two generations for each replicate population in the AF and CF populations. Although a small subset of variants (2.9%) showed allele frequency changes in the same direction in all three populations in the CFs, more than twice as many variants (6.4%) in the AF populations showed such reproducible allele frequency changes between the two generations (*P* < 2.2 × 10^−^^16^, chi-square test). Moreover, compared with the CF populations, many more variants demonstrated reproducible allele frequency changes of higher magnitudes in the AF populations (fig. 2*B*), indicating that hypoxia-specific selection was much stronger than genetic drift or hypoxia-independent natural selection in the laboratory.

To locate the variants responding to hypoxia-specific selection, we compared allele frequency differences between the AFs and CFs in the 17th generation using a generalized linear mixed model (GLMM, see Materials and Methods). This approach identifies correlated allele frequency differences between the two treatments at each of the 1,075,949 variant positions by taking into account the heterogeneity in sequencing depth between populations and fluctuations in allele frequencies among replicate populations within each treatment. The GLMM provides a *P* value for each variant in the data set and extreme *P* values indicate highly correlated allele frequency differences between the two treatments. To rule out that the extreme *P* values are due to stochastic differences arising from sampling errors, genetic drift, and heterogeneity in sequencing depth, we performed GLMM on nine additional data sets generated by permuting the labels of the six replicate populations in the 17th generation. None of these nine data sets generated *P* values as extreme as those observed between the AFs and CFs (*P* < 2.2 × 10^−^^16^, chi-square test; supplementary fig. S2, Supplementary Material online).

**Fig. 3.**
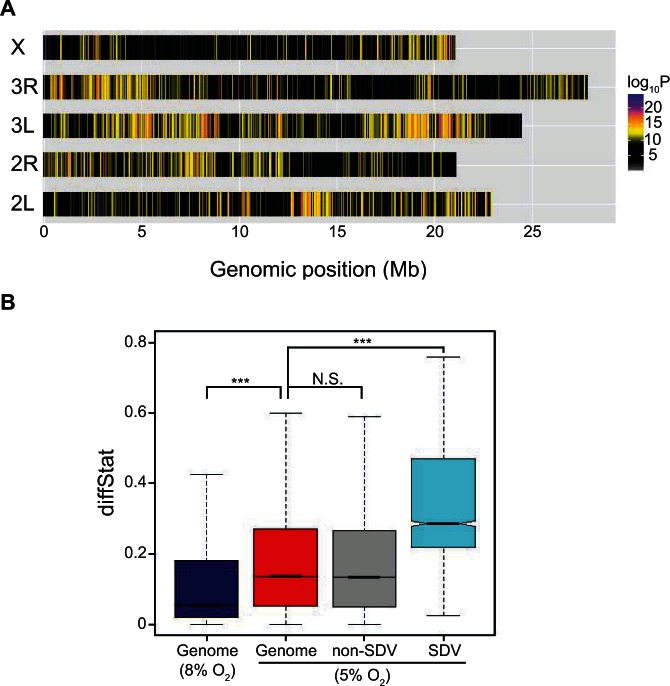
Distribution of SDV and diffStat scores. (*A*) Distribution of SDV in the five major chromosomal arms of the *Drosophila melanogaster* genome. Colors indicate the −log_10_(*P* value) obtained from the GLMM for each variant. (*B*) Comparison of whole-genome diffStat scores before (blue) and after the onset of hypoxia (red). Compared with the genomewide background and the nonsignificant variants (gray), the SDV (light blue) have much higher diffStat scores. Horizontal bars with notches indicate the median, the edges of the box indicate the interquartile range, and the whiskers indicate 1.5 times the interquartile range. ***P < 2.2 × 10^−16^, Wilcoxon rank sum test.

We compared the observed and permuted distributions to assess the false discovery rate (FDR) for genomewide significance (supplementary table S1, Supplementary Material online, also see Materials and Methods). Using a threshold of FDR <5%, we identified a total of 3,828 significantly diverged variants (SDV) corresponding to approximately 0.37% of all variants in the genome. A small fraction of the SDV (0.55%) were in heterochromatin and the dot chromosome, and were therefore removed from further analysis. No significant variants were observed in the mtDNA. The remaining 99.45% SDV (*n* = 3,806) were distributed throughout the five major chromosome arms of the *D. melanogaster* genome (fig. 3*A*). Very few, if any, of the SDV are expected to be mutations that arose after hypoxia selection because all of them are shared between the AF and CF populations and 95% are also present in the DGRP lines, indicating that adaptation occurred primarily through standing polymorphisms that are common and widespread in natural fly populations (supplementary fig. S1*B*, Supplementary Material online). A list of all the SDV, their coverage statistics, and gene annotations are listed in supplementary table S2, Supplementary Material online.

We further investigated each SDV by calculating “diffStat,” which is the smallest allele frequency difference in nine pairwise comparisons between the three replicate AF and CF populations (Turner et al. 2011). The higher the diffStat scores, the larger the allele frequency changes between the two treatments. The median diffStat score in the fourth generation was 5.64% (fig. 3*B*, dark blue), reaffirming our earlier observations that minimal allele frequency differences existed between the AF and CF populations prior to the sustained hypoxia selection (fig. 2). On the contrary, median diffStat after 17 generations of selection was 13.77% (fig. 3*B*, red). Importantly, the diffStat score for the SDV was significantly higher than that for the genomewide score in the 17th generation (*P* = 2.2 × 10^−^^16^, Wilcoxon rank sum test; fig. 3*B*, light blue). Such an increase in diffStat score was not observed for the nonsignificant variants (*P* > 0.05, Wilcoxon rank sum test; fig. 3*B*, gray). These results indicate that very few of the SDV are statistical false positives.

### Genetic Hitchhiking

**Fig. 4.**
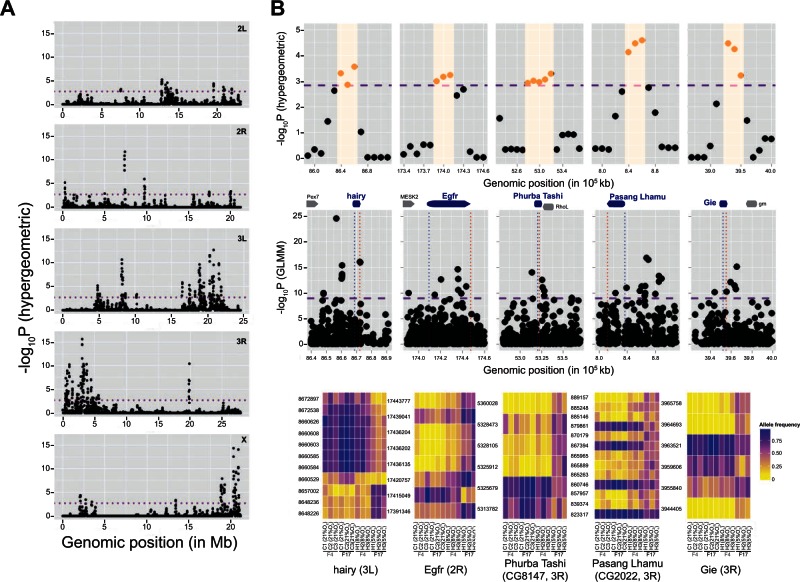
Candidate genomic regions under selection in AF. (*A*) Manhattan plots demonstrating clustering of SDV in certain genomic regions in the AF. Each dot represents a 50-kb sliding window with 10-kb steps. The dotted line shows the genomewide multiple testing threshold at FDR ≤5%. The *y* axes show negative log_10_ of *P* values obtained from the hypergeometric test and the *x* axes represent the genome size in Megabases. (*B*) Top panels: fine scale mapping of the genomic regions with clusters of SDV. Each dot represents a 50-kb sliding window. In each panel, orange region indicates a single differentiated block with multiple significant 50-kb windows. A differentiated block ±50-kb regions is shown in each panel. Middle panels: Differentiated block and surrounding regions from the corresponding panel in the top. Each dot represents a variant, *y* axis represents −log_10_(*P* value) obtained from the GLMM, and the dotted lines indicate the genomewide FDR <5% assessed using permutation tests. Genes in each region with at least one SDV are shown on top of each panel. Genes in blue are candidate hypoxia genes and the dotted lines in each panel indicate their location in that region. Bottom panels: Allele frequencies of the SDV in the corresponding middle panels in the AF and CF populations before and after hypoxia treatment. Each row is the SDV and each column is AF or CF population. Genomic co-ordinate of each SDV is listed on the left and the chromosomal arms and the candidate hypoxia genes harboring or adjacent to these SDV are listed on the bottom.

Although low FDR assessed by permutation testing assures that the probability of observing extreme *P* values by genetic drift or heterogeneity in sequencing depth is minimal, many of the SDV could be the result of genetic hitchhiking by nonselected variants that are in linkage disequilibrium with variants targeted by selection (Smith and Haigh 1974). It is difficult to pinpoint the hitchhiking SDV in pooled sequencing data because individual haplotype information is not available. Hence, we attempted to identify genomic regions that have clusters of SDV. To do so, we divided each chromosome arm into genomic bins of various sizes and using sliding windows we tested whether each window has an excess of SDV compared with the chromosomal background. We scanned the genome in 50-, 100-, and 250-kb windows with step sizes of 10, 25, and 50 kb, respectively. A majority of the genomic regions with significant overrepresentation of the SDV were shared among all of the window sizes tested (supplementary fig. S3, Supplementary Material online). With the purpose of identifying candidate genes, we focused on 50-kb windows for further analysis (fig. 4*A*). After adjusting for genomewide multiple correction using FDR < 5% (see Materials and Methods for details), we identified 358 significant 50-kb windows that could be further consolidated into 66 nonoverlapping blocks (supplementary table S3, Supplementary Material online). The median size of the differentiated blocks was 90 kb and these blocks were separated from one another by a median distance of 325 kb. These blocks likely represent genomic regions containing SDV targeted by selection as well as linked SDV that underwent hitchhiking.

### Identification of Putative Targets of Hypoxia-Specific Selection

In total, the 66 differentiated blocks contain approximately 45% of all the SDV and these SDV were either within or near 377 genes. To identify candidate genes in hypoxia, we focused on the most significant variant within each block. We identified 66 most significant SDV and they were located within 85 genes (for intergenic SDV both the genes flanking it were considered). One of these 85 candidate genes was *tango* (*tgo*), or hypoxia-inducible factor *HIF-1β*, a classic hypoxia response gene.

To evaluate whether any other candidate genes might be biologically relevant in hypoxia, we determined which of the candidates are also differentially expressed between the CF and AF populations and examined their individual identities. Nineteen of the 85 candidate genes, harboring noncoding SDV, potentially in their regulatory elements, are differentially expressed between CF and AF larva that have adapted to 5% O_2_ (Zhou et al. 2008). These differentially expressed candidate genes include: *hairy* (*h*), *epidermal growth factor receptor* (*Egfr*), *Ecdysone-induced protein 74EF* (*Eip74EF*), *frizzled2* (*fz2*), *B4, Icarus* (*ics*), *skywalker* (*sky*), *pollux* (*plx*), *Transport and golgi organization* (*Tango13*), *GTPase indispensable for equal segregation of chromosomes* (*Gie*), *ACXC, alan shepard* (*shep*), *CG3819*, *CG4229*, *CG4365, CG9331*, *CG8147, CG17150*, and *CG32541* (previously known as *CG43759*). Among these genes *hairy* and *Egfr* are known to play important functional roles in hypoxia tolerance (fig. 4*B*) (Zhou et al. 2008; Xie et al. 2013).

### Additional Candidate Genes under Selection

**Fig. 5.**
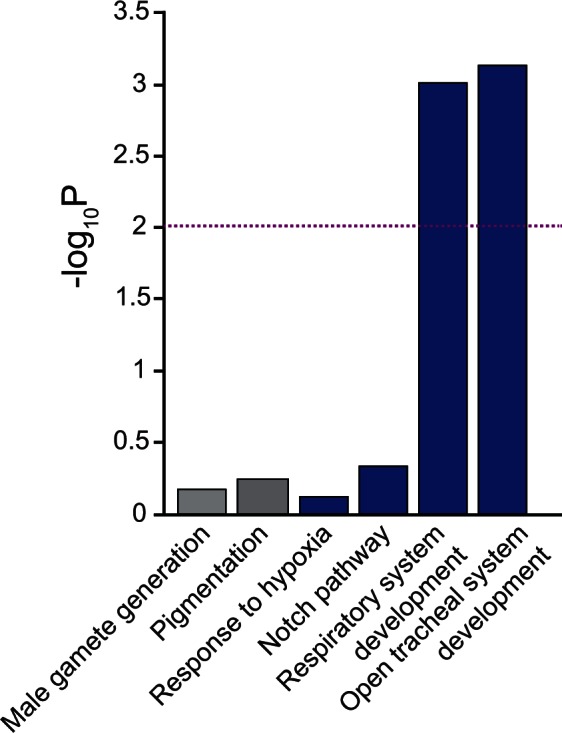
Enrichment of SDV for genes associated with hypoxia. Genes in hypoxia associated categories (blue) are enriched for SDV with extreme *P* values, whereas genes in control groups (gray) with no obvious functions in hypoxia are not. The *y* axis represents −log_10_ (*P* values) obtained from the enrichment analysis and the dotted line represents the Bonferroni adjusted *P* < 0.01 threshold for significance.

A large fraction of the SDV (55%) lies outside of the large blocks of linked variants. Most of these SDV are instead clustered over shorter genomic distances, likely in genomic islands representing shorter linkage. As an alternative to the linkage block approach, we evaluated whether the SDV with the most extreme *P* values are enriched for genes that are likely to be associated with hypoxia (fig. 5, see Materials and Methods for details). The most striking result was that these SDV were highly enriched for a set of genes common to the Gene Ontology (GO) terms “respiratory system development” and “open tracheal system development” (Bonferroni-adjusted *P* < 4.0 × 10^−^^03^ for both, permutation test). However, we found no enrichment for genes in “Response to hypoxia” category, perhaps because this GO category is poorly annotated in flies as many of the genes experimentally shown to respond to hypoxia (Dekanty et al. 2010; Azad et al. 2012) are not included in this GO term. Interestingly, we also did not find enrichment of the top SDV for genes in “Notch signaling” pathways (*P* > 0.05, permutation test). To test whether such enrichment could be observed by random chance, we performed the same analysis with genes that have no obvious functions in hypoxia, namely genes associated with pigmentation and male courtship behavior and observed no enrichment (*P* > 0.05, permutation test).

Based on these results, we identified 16 candidate genes associated with respiratory/tracheal system development that contain a SDV with extreme *P* values. Only two of these genes overlapped with the candidate genes that were present in the differentiated blocks. Combining these genes with the candidate genes in the differentiated blocks, we obtained a total of 99 genes that were further considered candidates for hypoxia tolerance in AF (supplementary fig. S4 and table S4, Supplementary Material online). Of the 99 candidate genes, 22 were also differentially expressed between larva of the AF and CF populations (supplementary table S4, Supplementary Material online).

### Knocking Down Expression of Candidate Genes Affects Development in Hypoxia

The *Drosophila* GAL4/UAS-RNAi system is an effective method to knockdown mRNA levels of a specific gene of interest (Fischer et al. 1988; Brand and Perrimon 1993). We used this system to evaluate the impact on hypoxia tolerance of six candidate genes for which RNAi lines were readily available (Dietzl et al. 2007). Four of the candidate genes tested, *plx*, *Gie*, *CG4365*, and *CG8147* harbor noncoding SDV and these genes are differentially expressed between AF and CF larva. *CG2022* contained several noncoding SDV but it was not differentially expressed between the AF and CF larva, likely because this gene is expressed in pupa and adults (Graveley et al. 2011) All five of these genes have little known functions in *D. melanogaster.* The sixth candidate gene we tested, *uninflatable* (*uif*) is associated with tracheal development and is upregulated in AF larva. However, its role in hypoxia has not been characterized. We selected these six candidate genes because they presented us with the opportunity to identify novel genes that are functionally relevant for hypoxia tolerance in flies.

**Fig. 6.**
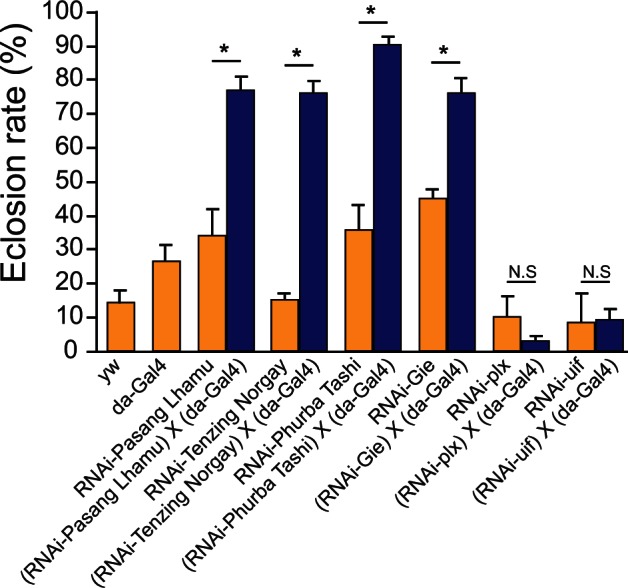
Functional validation of candidate genes. *Drosophila* stocks carrying UAS-RNAi transgenes targeting each candidate gene were crossed with da-GAL4 to knock down the expression of six candidate genes (blue bars). To ensure that response to hypoxia was not due to the genetic background carrying each transgene, we also scored the eclosion rates of flies with the general genetic background (yw), flies with Gal4 only, and lines carrying each UAS-RNAi construct (yellow bars). The progenies were cultured under hypoxic condition with 5% O_2_ to determine hypoxia tolerance of each cross. A significantly enhanced hypoxia tolerance was observed in the da-GAL4 crosses with UAS-RNAi-*Pasang Lhamu* (*CG2022*), UAS-RNAi-*Tenzing Norgay* (*CG4365*), UAS-RNAi-*Phurba Tashi* (*CG8147*), and UAS-RNAi-*Gie.* The da-GAL4 crosses with UAS-RNAi-*plx* showed marked decrease in hypoxia survival but statistical significance was not achieved because of low numbers. No difference in hypoxia survival was observed with da-GAL4XUAS-RNAi-*uif.* Each bar represents mean ± SEM of six replicate tests in two separate experiments. The significant genes with previously unknown functions were subsequently named after three Sherpa mountaineers known for their legendary ascents of Mount Everest. *P value < 0.01, Student’s *t*-test.

All of the *GAL4;UAS-RNAi* flies reared in normoxia developed normally with no obvious disturbances in development, demonstrating that downregulation of these candidate genes has no significant effect on development in normoxia. To test whether these genes affect developmental process in hypoxia we measured eclosion rate, which is the ratio of adult flies that have completed the pupal stage to those flies whose development was arrested, in each population. We observed that four of the six *GAL4;UAS-RNAi* lines exhibited increased hypoxia tolerance compared with controls at 5% O_2_ (fig. 6, blue and yellow bars, respectively). As three of these genes, *CG2022*, *CG4365*, and *CG8147*, do not have gene names in *D. melanogaster,* we hereafter refer to them as *Pasang Lhamu* (*CG2022*), *Tenzing Norgay* (*CG4365*), and *Phurba Tashi* (*CG8147*), honoring the legendary Sherpa mountaineers (fig. 6). These results demonstrate that the candidate genes we have identified indeed have the potential to underlie the adaptive hypoxia response.

### Shared Positively Selected Genes between Flies and High-Altitude Human Populations

As oxygen is critical for cellular functions in most eukaryotes, genes associated with respiratory system development and cellular metabolic mechanisms that generate energy from oxygen are highly conserved (Rytkonen et al. 2011). Many of the genes involved in respiratory development in flies are also associated with lung development, vasculature development, and angiogenesis in mammals. Because both categories of genes were identified in our study, we explored whether orthologs of genes that are under selection in the AF populations might also be under positive selection in high-altitude human populations. To do so, we first obtained population branch statistics (PBS) values from whole-genome SNP genotype data from previously published studies for four high-altitude human populations, namely Andeans (*n* = 10), Sherpas (*n* = 69), Tibetans (*n* = 96), and Ethiopian highlanders (*n* = 165) (Alkorta-Aranburu et al. 2012; Zhou et al. 2013; Jeong et al. 2014). PBS is a cross-population measure of allele frequency change at a given locus in a population since its divergence from two other populations. In comparison between a high-altitude population and two low land populations, a large PBS value at a particular SNP represents a marked divergence of allele frequency at that locus in the high-altitude population relative to two low land populations. One possible cause of such divergence is positive selection. This approach has been widely used to identify candidate genomic loci under positive selection in several high-altitude human populations (Yi et al. 2010; Alkorta-Aranburu et al. 2012; Huerta-Sanchez et al. 2013; Zhou et al. 2013; Udpa et al. 2014).

**Fig. 7.**
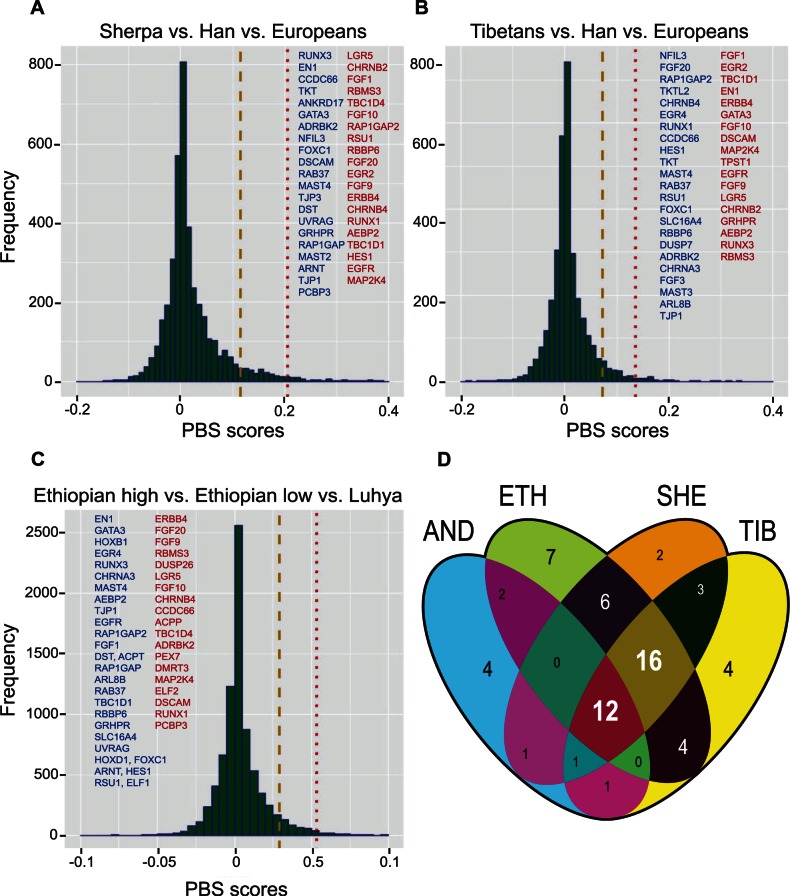
Shared genes under positive selection. Genomewide distribution of PBS scores in Sherpas (*A*), Tibetans (*B*), and Ethiopian highlanders (*C*). Top 5% and 1% tails of the distribution are indicated by the dashed yellow and the dotted red lines. Human orthologs of candidate genes in AFs that have at least one SNP in the top 5% and 1% tails are shown in blue and red, respectively. 41, 41, and 47 positively selected genes were identified in the Sherpas, Tibetans, and Ethiopians, respectively; and 28 are shared between all three human populations (*D*). Twelve of the 28 shared genes also harbor markedly divergent SNPs in Andeans (*D*).

Of the 99 *Drosophila* candidate genes under selection in the AF populations, 55 genes have 102 putative orthologs in humans (supplementary fig. S4, Supplementary Material online). We evaluated whether these 102 genes are significantly enriched for SNPs with large positive PBS values in each of the four high-altitude populations. Remarkably, human orthologs of the candidate genes in flies were strongly enriched for highly differentiated SNPs in three human populations (top 5% PBS tails, *P* = 2.1 × 10^−^^07^, 1.6 × 10^−^^02^, and 1.4 × 10^−^^04^, binomial test, in Sherpas, Tibetans, and Ethiopian highlanders, respectively; fig. 7*A*–*C*). These genes were also significant for the extremely differentiated SNPs in these high-altitude populations (top 1% PBS tails, *P* = 1.73 × 10^−^^05^, 7.9 × 10^−^^04^, and 2.7 × 10^−^^03^, binomial test, in Sherpas, Tibetans, and Ethiopians, respectively; fig. 7*A*–*C*). A total of 41, 41, and 47 genes contained at least one SNP in the top 5% PBS tails in Sherpas, Tibetans, and Ethiopians, respectively (supplementary table S5, Supplementary Material online). More interestingly, 28 of these genes were shared among the three high-altitude human populations (fig. 7*D*). However, we did not observe an enrichment for highly divergent SNPs for the candidate genes in the Andeans (*P* > 0.05, binomial test), likely because of very small sample size in this study (Zhou et al. 2013). Nevertheless, 21 candidate genes harbored SNPs in the top 5% PBS tail in Andean highlanders (supplementary table S5, Supplementary Material online) and 12 of these genes were shared among all four high-altitude populations (fig. 7*D and *supplementary table S6, Supplementary Material online). Furthermore, human orthologs of candidate genes in flies are highly enriched for SNPs that are in the extreme 5% PBS tails in all four high-altitude populations (*P* = 3.5 × 10^−^^03^, binomial test).

## Discussion

Darwin (1858) argued that individuals in a population harbor “varieties” and these subtle variations can be advantageous in a new environment. We now realize that upon sudden change of environment, previously neutral and readily available standing variation underlying such subtle phenotypic differences can become beneficial. Using experimental evolution and pooled sequencing, we identified several thousand common widespread standing variants that responded to selection in a severe hypoxic environment in replicate populations. Although we were unable to use haplotype-based tests due to the nature of pooled sequencing data, such tests that scan the genome for selective sweeps may not be appropriate in detecting signals of selection in experimentally evolved populations for several reasons. First, standing variation is likely to be distributed in multiple haplotypes and when acted upon by selection, they can change in allele frequencies without dramatic loss of genetic diversity at linked sites (Hermisson and Pennings 2005; Burke 2012; Jha et al. 2015). Second, selection was conducted for a short term (17 generations) and most SDV responding to hypoxia selection, including the one within the differentiated blocks, did not reach fixation. Thus, selective sweeps are likely to be rare in experimentally evolved populations. Indeed, selective sweeps were rare in the AF even after 180 generations of hypoxia selection (Zhou et al. 2011). Moreover, the handful of selective sweeps that were identified after such prolonged period of hypoxia selection was in genomic regions distinct from the differentiated blocks that we identified in the 17th generation, indicating that they may have resulted from selection operating on a rare or beneficial de novo mutation that occurred in later generations.

Large numbers of significant variants distributed throughout the genome, as we have observed between the AF and CF populations, is a common feature in experimental evolution studies (Turner et al. 2011; Orozco-terWengel et al. 2012; Remolina et al. 2012; Turner and Miller 2012; Cassidy et al. 2013; Tobler et al. 2014; Jha et al. 2015). Allele frequency divergence in experimental populations can have disparate causes, including the trait-specific selection, background selection for fitness in the cage environment, genetic drift, and genetic hitchhiking. Comparison of the replicate AF and CF populations in the 17th generation should minimize the contributions of background selection as flies from both treatments were maintained in identical cage conditions. Low FDR (<5%) based on permutation testing indicates that the effects of genetic drift or heterogeneity in sequencing depth are also negligible in our study. Even though linkage disequilibrium in nonheterochromatin regions extends to 10–200 bp in *D. melanogaster* (Mackay et al. 2012), a certain amount of long-distance hitchhiking can be expected in the AF populations because of the small numbers of isogenic lines used to establish the founding population (Nuzhdin and Turner 2013; Tobler et al. 2014). Therefore, given such large numbers of significant variants, the challenge is to distinguish the SDV that are targets of hypoxia-specific selection from those resulting from hitchhiking and/or unintended natural selection in the laboratory. We employed several filtering approaches to minimize the numbers of nonhypoxia-related SDV. First, using a clustering approach we identified SDV hitchhiking in large differentiated genomic blocks averaging 90 kb in size. Second, we prioritized the genes harboring the most significant variants within each block. Finally, we used a GO enrichment analysis to identify additional SDV in genes that are relevant to hypoxia tolerance. In total, we were able to identify 99 candidate genes that have diverse biological functions that are not limited to oxygen sensing and transport.

Twenty-two of the 99 candidate genes we identified were also differentially expressed between larva of AF and CF populations and some of them have well-defined functions in hypoxia resistance in flies. For example, one of the candidate genes, *hairy,* is a known hypoxia response gene in flies. As our prior results have demonstrated, it plays a key role in metabolic regulation during hypoxia (Zhou et al. 2008). Metabolic rewiring is considered one of the hallmarks of hypoxia response and it is essential for cellular survival in hypoxia (Hochachka et al. 1996; Bigham et al. 2014). Another candidate gene, *Egfr* activates Ras-raf-MAPK signaling pathway—which is also vital in hypoxia. Both *hairy* and *Egfr* are also involved in respiratory system development and influence Notch signaling, which in turn plays an important role in hypoxia tolerance in flies (Zhou et al. 2011). Several of the candidate genes that were not differentially expressed in larva are highly expressed in embryos or pupa (Graveley et al. 2011). As mRNA expression from these developmental stages is not available, the individual contributions of these genes in hypoxia remain to be determined. Furthermore, as a proof-of-concept, we also used RNAi-mediated gene expression knockdown of a handful of candidate genes. Our results revealed several novel candidate genes, such as *Pasang Lhamu*, *Tenzing Norgay*, and *Phurba Tashi* that are functionally relevant in hypoxia tolerance in the AF populations. These results increase our confidence in the functional relevance of the candidate genes and future work with additional RNAi lines may reveal the functional relevance of many of the candidate genes that we have identified in hypoxia response in flies.

Interestingly, several orthologs of candidate genes that we identified in flies also harbor markedly differentiated SNPs in four geographically distinct high-altitude populations that have adapted to low oxygen. Eight of the 12 genes shared between all four high-altitude human populations are either directly regulated by HIF or functionally relevant to hypoxia (supplementary table S6, Supplementary Material online). For example, *EGFR* is a direct target of HIF-1 (Wang et al. 2007; Schodel et al. 2011). *EGFR*, along with *RBMS3* is induced by hypoxia in human cell culture experiments (Wang et al. 2007; Satoh et al. 2015). The *Drosophila* orthologs of both these genes are also upregulated in the larva of the AF populations (supplementary table S4, Supplementary Material online) (Zhou et al. 2008). Similarly, *FGF10* and *ERBB4* increase HIF-1α activity in humans (Scott et al. 2010; Paatero et al. 2012). Moreover, *AEBP2* is associated with pulmonary function (Soler Artigas et al. 2011) and *GATA3* is associated with serum calcium levels (O’Seaghdha et al. 2013) in previous genomewide association studies (Welter et al. 2014). As these genes harbor SNPs with marked allele frequency divergence in geographically distinct high-altitude human populations as well as in three independently evolved replicates of *D. melanogaster* populations and have plausible functions in hypoxia, they are likely to be under positive selection in response to hypoxia in high-altitude human populations. Although these genes serve as excellent candidates, additional work is required to pinpoint the variants that are targeted by selection in each population and to elucidate the mechanisms by which they affect hypoxia tolerance.

Our findings contrast with previous studies in high-altitude human populations. Although we identify over 100 candidate genes in three replicated hypoxia-adapted *Drosophila* populations and several candidate genes shared between geographically distinct high-altitude human populations, many of the positively selected genes in high-altitude humans have been reported to be unique to a particular population (Bigham et al. 2010; Alkorta-Aranburu et al. 2012; Scheinfeldt et al. 2012; Huerta-Sanchez et al. 2013; Eichstaedt et al. 2014). For example, despite being under positive selection in Himalayan populations, signals of selection were not detected in *EPAS1* in Andeans or Ethiopians residing at high altitudes (Bigham et al. 2010; Alkorta-Aranburu et al. 2012). Similarly, *EGLN1* appears to be under positive selection in Himalaya and Andes but not in Ethiopia (Bigham et al. 2010; Alkorta-Aranburu et al. 2012), whereas *BHLHE41* appears to be under selection only in Ethiopia (Huerta-Sanchez et al. 2013). There is no doubt that locale-specific signals of adaptation have occurred due to founder effects, de novo beneficial mutations that arose in each population, and introgression of DNA carrying beneficial mutations from nearby populations (Huerta-Sanchez et al. 2014; Jeong et al. 2014). However, very little overlap of candidate genes across high-altitude populations has been puzzling.

The shared candidate genes that we have identified may have remained undetected in previous studies for a variety of reasons. Methods of detecting signatures of selection vary between different high-altitude human studies. Some high-altitude human studies have attempted to locate genomic regions with highly reduced heterozygosity (Simonson et al. 2010; Eichstaedt et al. 2014). As a large reduction in heterozygosity results from selection acting on rare or de novo mutations, these studies have likely discovered only a small subset of genes that are under selection in high-altitude humans (Hermisson and Pennings 2005; Hernandez et al. 2011). Other studies have identified several hundred SNPs that show marked allele frequency differences in high-altitude populations (Bigham et al. 2010; Yi et al. 2010; Alkorta-Aranburu et al. 2012; Huerta-Sanchez et al. 2013; Zhou et al. 2013; Udpa et al. 2014). However, pinpointing those variants are likely due to positive selection in response to hypoxia has been difficult. As a result, most studies have focused either on a small subset of highly differentiated SNPs or on a subset of candidate genes with known functions in hypoxia (Bigham et al. 2010; Simonson et al. 2010; Alkorta-Aranburu et al. 2012; Jeong et al. 2014). Hence, these studies likely have missed those genes that have little known functions in hypoxia. Moreover, a variety of other parameters such as differences in methods of detecting allele frequency differentiation, ascertainment biases in SNPs due to the differences in genotyping platforms, variation in sampling altitudes, the choice of low-land control and outgroup populations, unknown demographic histories of each high-altitude populations, and differences in genomewide significance thresholds may have contributed to the lack of reproducibility between studies. For instance, Bigham et al. (2010) and Zhou et al. (2013) identified large numbers of differentiated variants in high-altitude Andeans. As the genotyping platforms and methods to detect selection differed between these two studies, each study nominated a distinct set of candidate genes for positive selection, even though both included individuals from Cerro de Pasco in Peru. It is important to note that the signals of selection not only differ between studies but also vary within the same data set, depending upon the test for positive selection (Bigham et al. 2010; Simonson et al. 2010; Zhou et al. 2013).

It is also noteworthy that the limited sample sizes used in most of the published studies may have made it difficult to identify all of the signals of selection in high-altitude human populations. Although some tests of selection, such as XP-EHH (Sabeti et al. 2007) can detect signals of selection with limited sample sizes, others such as iHS (Voight et al. 2006) require a larger sample size (Pickrell et al. 2009). However, both of these tests are limited to detecting selective sweeps that are at moderate frequencies (Pickrell et al. 2009). F_ST_ outlier-based tests, such as PBS, can detect signals of positive selection even if selection does not manifest into selective sweeps (Yi et al. 2010). However, small sample sizes may increase the coefficient of variation of genomewide F_ST_ estimates obscuring the true outliers (Willing et al. 2012). For example, the Ethiopian data set we used here had the largest sample size (*n* = 165) and also the largest number of orthologous genes that are enriched for highly divergent SNPs (*n* = 47); whereas, Andeans, the population with smallest sample size (*n* = 10), showed no enrichment. Moreover, many human orthologs of candidate genes in flies that are shared between Sherpas, Tibetans, and Ethiopians and are also associated with HIF-1 or EPAS1 activity in humans were not shared with Andeans (supplementary table S6, Supplementary Material online). With larger sample sizes additional genes that share hallmarks of positive selection will likely be revealed across all high-altitude human populations.

Finally, even though large numbers of differentiated variants have been identified between high-altitude and lowland populations, it has been difficult to identify SNPs that have specifically responded to hypoxia. For example, local environmental factors such as temperature, diet, and pathogens are known to have exerted strong selective pressures throughout human evolution (Perry et al. 2007; Hancock et al. 2010; Fumagalli et al. 2011). Indeed, the strongest signals of selection in some high-altitude human populations are in genes associated with immunity and inflammatory responses (Bigham et al. 2010; Alkorta-Aranburu et al. 2012), indicating that the strength of selection exerted by local pathogens may be stronger than that due to hypoxia in geographically distinct high-altitude regions. As a result, almost all studies have focused either on HIF-regulated genes with well-known functions in hypoxia (Moore et al. 2004) or on the top 0.1–0.2% variants in the genome (Alkorta-Aranburu et al. 2012; Huerta-Sanchez et al. 2013). However, very small proportion of the positively selected candidate genes in high-altitude humans such *EPAS1* and *EGLN1* are in the HIF pathway (Bigham et al. 2010; Simonson et al. 2010; Alkorta-Aranburu et al. 2012) and some positively selected genes in high-altitude humans with functions in hypoxia tolerance are likely HIF independent (Zhou et al. 2013; Udpa et al. 2014).

In contrast to natural populations, hypoxia is likely the strongest selective pressure in the experimentally evolved populations. Hence, the majority of the candidate genes is likely hypoxia specific. Enrichment of highly divergent SNP in orthologs of these genes in high-altitude human populations, many of which have known functions in hypoxia, further suggests that these genes are likely under positive selection in multiple high-altitude human populations. It is noteworthy, however, that several additional genes, in addition to the candidate genes we have identified, are likely under positive selection in high-altitude human populations. These genes were likely not identified using our approach, partly due to the physiological differences between humans and flies. Additionally, in this study, we focused on comparing the PBS signals across the high-altitude human populations. Comparing signals from other tests of selection may reveal additional positively selected genes that are shared across high-altitude human populations (Scheinfeldt et al. 2012; Xing et al. 2013; Wuren et al. 2014).

In conclusion, we have used experimental evolution in *D. melanogaster* to show that hypoxia tolerance is a highly polygenic trait that involves over 100 genes in diverse biological and molecular processes. Many of these genes are also under positive selection in multiple high-altitude human populations, indicating that fundamental genetic mechanisms regulating hypoxia tolerance have remained conserved throughout evolution. More strikingly, these results indicate that convergent evolution can occur between organisms hundreds of millions of years diverged through selection acting on standing variation in conserved genes that contribute to an ancient complex trait.

## Materials and Methods

### *Drosophila melanogaster* Populations

The selection scheme and the phenotypes of the flies have previously been described in detail (Zhou et al. 2007, 2008). Briefly, 27 isofemale lines descended from individual wild-caught *D. melanogaster* females (kindly provided by Dr Andrew Davis) were used to create a single laboratory cage population (founding population) with 20 males and 20 virgin females from each line (1,080 flies in total). Isofemale lines are populations of flies that are derived from a single mated female. As the isofemale lines used in our population were caught in the wild, it is highly likely they were inseminated by multiple males. Hence, each line carries a minimum of four haploid genomes initially, depending on whether they are singly or multiply mated. Thus, considerable genetic diversity existed in the founding population.

Embryos collected from this founding population were used to establish six replicate subpopulations, three of which were subjected to hypoxia (AF1, AF2, and AF3) in population chambers connected to 8% O_2_ balanced with nitrogen. The three control populations (CF1, CF2, and CF3) were grown in normoxia in similar chambers at 21% room oxygen levels. Each replicate population was maintained at relatively large population size (over 2,000 flies per population). Hypoxia selection was initiated at 8% O_2_, a level that had little impact on survival rates, and O_2_ concentration was subsequently reduced every 3–5 generations by 1%. At no point, did a reduction in O_2_ concentration cause a severe population decline or bottleneck. By generation 13 AF flies were able to complete development and maintain populations at 5% O_2_. Adult flies from the 17th generation (5% O_2_) were collected and stored (−80 °C) for DNA extraction.

### DNA Extraction, Sequencing, and Alignment

DNA was extracted individually from 100 flies per replicate population (1,200 individuals in total from 12 replicate populations) using DNeasy Blood and Tissue kit (Qiagen). Individual fly was placed in a tube on ice and pulverized using a mortar and pestle in presence of 180 μl ATL buffer. This mixture was incubated at 56 °C on a mixer for 4 h in presence of 20 μl proteinase-K. Subsequent extraction procedure was performed according to the manufacturer’s protocol. DNA concentration was measured using Nanodrop and equal amount of DNA from all 100 flies per replicate population was mixed to create a total of six pooled-DNA samples each containing 200 chromosomes from 100 flies. Each pooled-DNA sample was fragmented to 500 bp using a Covaris sonicator. DNA fragments were size selected, and Illumina paired-end libraries were generated from each of the 12 populations at the High-throughput Genomic Analysis Core Facility of Institute of Genomic and Systems Biology at The University of Chicago. Each library was first sequenced in a single lane of Illumina HiSeq2000 producing 50-bp single end reads. A 100-bp paired-end sequencing was performed in multiple lanes.

Data analysis was performed on the Bionimbus cloud (www.bionimbus.org), an Open Science Data Cloud project focused on biological data, especially Next Generation Sequencing data. FastX toolkit (http://hannonlab.cshl.edu/fastx_toolkit/) was implemented to check the quality of each read. Reads were mapped to the *D. melanogaster* reference genome (dm3, BDGP Release 5) using BWA (Li and Durbin 2009). GATK (McKenna et al. 2010) was used for indel realignment and base quality recalibration. Alignments with mapping qualities less than 15 were discarded, and Samtools –rmdup (Li et al. 2009) was used to remove potential polymerase chain reaction duplicates. Depth of coverage was calculated using BedTools (Quinlan and Hall 2010). Each population was sequenced at 26–99× median sequencing depth. In total, 95–99% of the genome was covered over 10×, 76–98% of genome was covered above 20×, with only 1% of the reference genome not represented.

### Genotyping and Identification of High-Confidence Variants

Variant calling was performed as described previously (Cassidy et al. 2013) because this method has minimal FDR (<2%). We first merged the bam files from all 12 populations to create a variant reference file (VarRef) with median sequencing depth greater than 1,000×. Such high sequencing depth allows for more accurate identification of polymorphic loci in the genome. Samtools -pileup (Li et al. 2009) was used on the VarRef as well as each bam file to extract information, such as base quality, mapping quality, and SNP quality at each chromosomal position. Custom perl scripts were used to parse pileup files and call polymorphisms.

A total of 32,397,996 polymorphic positions were identified by samtools pileup in the VarRef. Most of these variants were either singletons or present at negligible frequencies and were potential artifacts of sequencing error. To filter sequencing errors we discarded reads with Mq < 40. Assuming that 75% of the tri-allelic positions are due to sequencing error, we set the threshold for sequencing error at the 75th quantile in the read count distribution of the third allele position in the VarRef. In our case, this read count threshold was 3. Tri-allelic positions where third allele count was more than 3 across all populations were removed from further analysis and only those bi-allelic positions where the minor allele count exceeded three reads were considered. Only those positions with global sequencing depth over 10× were further analyzed. Over 7M bi-allelic positions passed these criteria. Removing highly (above the 95th quantile at bi-allelic sites) and lowly (less than 10× in each of the six populations) covered variants reduced total bi-allelic positions to approximately 4M. To take coverage differences between populations into account and to identify high-confidence polymorphisms, we calculated 99.73% binomial confidence interval for the minor allele at each variant position. Variants whose minor allele had lower bound 95%CI above zero in any one of the 12 populations were considered high-confidence polymorphisms. A total of 1,075,949 polymorphic positions relative to the reference sequence passed all the quality control procedures and were further analyzed. These variants included 96% SNPs (*n* = 1,032,642) and 4% indels (*n* = 43,307) that were used in subsequent analysis.

### Allele Frequency Divergence and the GLMM

Allele frequencies were estimated as a ratio of variant counts to coverage. Allele frequency differences between the 4th and the 17th generations were calculated as average[abs(*P*_i17_ − *P*_i4_)], where *P* indicates AF or CF and *i* indicates the replicate populations 1, 2, and 3. Such methods for quantifying selection induced changes in allele frequencies from pooled-sequencing data have a high false positive rate because variance in allele frequencies between populations due to genetic drift is not accounted, errors in allele frequency estimates due to varying sequencing depth in each sample are ignored, and adaptation due to hypoxia-independent selective forces in the laboratory-caged population is unaccounted.

In pooled sequencing, the basic statistics are the read counts at each variant position, treatment-groups (hypoxia vs. controls), and the number of replicate populations within each treatment. As these types of data are not expected to be normally distributed, traditional normality-based tests are not useful (Bolker et al. 2009). A GLMM to quantify association between the treatment groups and each of the 1,075,949 variant alleles was implemented using the R package “lme4.” For each variant, read count (reference = 0, variant = 1) was the outcome variable. Read count at bi-allelic positions was assumed to be binomially distributed, and treatment groups (hypoxia-tolerant and normoxic controls) were specified to have fixed effects whereas each replicate population (*n* = 6) was specified to have random effects. A *P* value for each variant was computed using the default method in the lme4 package. We note that the use of read counts and not estimated allele frequencies prevents false positives due to coverage differences at each variant position; however, GLMM essentially quantifies correlated allele frequency divergence in all three replicates. To assess whether such *P* values can be generated by drift or by chance alone, we generated all nine combinations of six populations by permuting their labels which did not disturb the underlying linkage distribution in each population and the above model was implemented on each combination. At various thresholds of *P* values (*P*), FDR was assessed by calculating the ratio of average numbers of variants (*s_n_*) that exceed the threshold in the permuted samples (*n*, where *n* = 1, 2, . . . , *N* = 9) to that in observed samples (*s_obs_*) and FDR < 5% was considered genomewide significance threshold.

$$\text{FDR}=\frac{R_{exp}}{R_{obs}}=\frac{\frac{1}{N}\sum_{n=1}^{9}(s_{n}<P\;)}{s_{obs}<P}.$$

The FDR thus calculated is very stringent because only nine permutations could be performed from six replicates. This FDR < 5% threshold is equivalent to FDR = 5 × 10^−^^07^ calculated using *q*-value (Dabney et al. 2012).

To calculate diffStat statistic, we first calculated change in allele frequencies between all nine possible combinations of AF and CF populations. We then identified variants that have changed in consistent directions in all nine comparisons. Then, we calculated the minimum absolute change in allele frequency difference between the two treatments as the diffStat score, that is, abs(min(AF1-CF1, AF1-CF2, AF1-CF3, AF2-CF1, AF2-CF2, AF2-CF3, AF3-CF1, AF3-CF2, AF3-CF3)).

### Hypergeometric Test for Identification of Differentiated Blocks

To test whether the SDV are overrepresented in certain genomic windows, we performed a hypergeometric test for 100-, 50-, and 10-kb sliding windows with 25-, 10-, and 2-kb step size in each major chromosome arm as follows: The total number of variants (*n*) and SDV (*s*) in each window were assessed. Given the total numbers of variants (*N*) and total number of SDV (*S*) in each chromosome arm, we computed the probability (*P*) of observing *s* SDV when *n* variants are sampled. For multiple-testing adjustment, *q*-values were estimated as above and FDR < 0.05 was used as the genomewide multiple testing threshold.

### Annotation of Polymorphic Loci and Enrichment Analysis

Gene annotation of polymorphisms was performed using ANNOVAR (Wang et al. 2010). Polymorphisms in intronic, exonic, or untranslated regions were annotated to a single gene. For intergenic variants, genes immediately upstream or downstream were considered.

To perform GO enrichment analysis for the SDV, we obtained genes included in several GO terms form FlyBase (St Pierre et al. 2014). We first identified four sets of genes in the GO database that are relevant for hypoxia tolerance in flies: Genes associated with “Response to hypoxia” (GO:0001666, *n* = 58 genes), “Respiratory system development” (GO:0060541, *n* = 324), and “Open tracheal system development” (GO:0007424, *n* = 304 genes). We compiled a list of 128 genes by including genes associated with “Regulation of Notch signaling pathway” (GO:0008593) and “Notch signaling pathway” (GO:007219) and refer to this set as “Notch pathway.” We then used GOWINDA (Kofler and Schlotterer 2012) to test whether genes in each of the four categories have overrepresentation of SDV in the top 5% tail of an empirical null distribution created by identifying the variant with the smallest *P* value (from the GLMM) for all other genes in the data set. GOWINDA simulates large sets of randomly sampled SNP to calculate *P* values for enrichment of each GO term (Kofler and Schlotterer 2012). Such permutation-based approach reduces biases associated with gene length and reduces false positive GO terms (Kofler and Schlotterer 2012). We conducted 100,000 simulations for each of the GO terms using gene annotations based on *D. melanogaster* reference genome version 3.0 and full coding region ±500-bp flanking regions were considered (–gene-definition updownstream500 and –mode gene were used in GOWINDA). Bonferroni correction was used to correct for multiple testing. As controls, we downloaded genes associated with “Male gamete generation” (GO:0040007, *n* = 258 genes) and “Pigmentation” (GO:0043473, *n* = 100 genes) as they have similar numbers of genes as in the four categories above.

### Functional Validation of Candidate Genes Using RNAi

The UAS-RNAi stocks were obtained from Vienna Drosophila RNAi Center (Vienna, Austria) with stock number 1047 (UAS-RNAi-uif), 2892 (UAS-RNAi-CG8147), 6261 (UAS-RNAi-CG4365), 9049 (UAS-RNAi-CG2022), 26085 (UAS-RNAi-Gie), and 27335 (UAS-RNAi-plx). The da-GAL4 driver (stock number 8641) was obtained from Bloomington *Drosophila* Stock Center (Bloomington, IN). All stocks were maintained on standard-cornmeal *Drosophila* medium at room temperature and 30–50% humidity. Virgin female of the da-GAL4 driver was crossed with male UAS-RNAi stocks individually. Each cross contained ten virgin females and ten males. The crosses were allowed to lay eggs for 48 h, and the eggs were then transferred to a computer-controlled atmospheric chamber and cultured under 5% O_2_ hypoxic condition for 3 weeks. At the end of the experiment, the number of empty and full pupae were counted. The ratio of empty to the total number of pupae was calculated and presented as eclosion rate. All the stocks and crosses were also cultured under room air condition with standard cone mile medium as normoxic controls. All crosses were performed in triplicates in two separate experiments. The statistical significance of survival hypoxia between each cross and control was calculated by Student’s *t*-test. *P* values < 0.05 were considered statistically significant.

### Identification of Human Orthologs and Fly–Human Hypoxia Gene Overlaps

Three algorithms were implemented to identify human orthologs of all approximately 13,000 *D. melanogaster* genes. Orthodb (Waterhouse et al. 2012) identified 8,685 human orthologs of 6,726 *Drosophila* genes. In total, 4,761 human orthologs of 5,029 fly genes were identified by NCBI’s Homologene (http://www.ncbi.nlm.nih.gov/homologene/) and 9,961 human orthologs of 7,857 fly genes were identified by modENCODE (http://compbio.mit.edu/modencode/orthologs/modencode-orths-2012-01-30/). From the modENCODE orthologs list, genes with ≥2:1 orthologs between fly and humans were removed and a total of 5,059 genes with 8,203 human orthologs were retained. Many of the obvious fly–human orthologs were missed by each of these algorithms; hence, we took the union of the three methods and removed ortholog calls that were conflicted by any one of those methods. In total, 7,311 human orthologs of 7,076 fly genes were identified. This is a pretty conservative estimate of total number of fly–human orthologs. Of the 99 genes under selection in flies, 55 genes had 102 orthologs in humans.

To identify genomic regions putatively under selection, we obtained the PBS values for four high-altitude human populations. We are limited to using PBS as a test for allele frequency divergence because the raw genotyping data for all these populations are not available to us. PBS data from Ethiopian individuals were obtained from Alkorta-Aranburu et al. (2012) in which the Ethiopian highlanders comprised 102 Amhara and 63 Oromo individuals residing at 3,700 and 4,000 m, respectively. Sixty Amhara residing at 1,200 m and 35 Oromos residing at 1,560 m were collectively used as low-altitude Ethiopian populations, and Luhya was used as the outgroup. PBS values from 69 Sherpa individuals residing at 3,800 m and 96 Tibetan individuals residing over 3,000 m (available from Jeong et al. [2014]) were calculated separately using HapMap Han Chinese as lowland populations and Europeans as the outgroup following the procedures described in Alkorta-Aranburu et al. (2012). We also obtained PBS values for Andeans individuals from Zhou et al. (2013) in which ten Andeans from Cerro de Pasco, Peru residing at 4,300 m were sequenced. PBS values for these individuals were calculated using 50-kb genomic windows with 2-kb step sizes, as described in Zhou et al. (2013), using 66 Mexicans as lowland controls and 67 Luhya included in 1000 Genomes Project Consortium et al. (2010). PBS was calculated for 277,553 SNPs in Sherpas, 279,386 SNPs in Tibetans, 551,334 SNPs in the Ethiopians, and 1,452,740 regions in the Andeans. The differences in the number of loci for which PBS could be calculated are due to differences in genotyping platforms used for each of these three populations.

Genomic loci at the extreme tails of the PBS distribution represent the most divergent regions between a high-altitude population and lowland controls. Considering genomic loci at the extreme 5% tail of the PBS distribution, we identified 13,867 SNPs in Sherpas, 13,945 in Tibetans, 27,483 in Ethiopians, and 13,867 regions in Andeans. These loci could have diverged due to genetic drift, nonhypoxia-related selection, and hypoxia-specific positive selection. To identify genetic loci that are likely responding to hypoxia-specific selection, we used the 102 human genes whose fly orthologs were putatively under selection in AF populations as candidate genes. Over representation of markedly divergent SNP in these genes in four independently evolved high-altitude human populations would be a strong indication that these genes are under hypoxia-specific positive selection. To test whether these candidate genes are enriched for markedly divergent SNP, we identified SNP within or adjacent to each candidate gene in each high-altitude population. In total, 3,158, 3,158, and 6,150 SNPs and 14,186 regions were located in the 102 candidate genes in Sherpas, Tibetans, Ethiopians, and Andeans, respectively. We then evaluated whether these SNP are enriched, relative to all other SNP or regions in the data set, in the top 5% and 1% tail of the PBS distributions in each of the four high-altitude populations. Statistical significance for the enrichment was assessed using a two-sided binomial test and *P* values < 0.05 were considered statistically significant. In total, 223, 185, and 371 of these SNPs were in the top 5% tails and 61, 51, and 85 were in the top 1% tails in Sherpas, Tibetans, and the Ethiopian highlanders, respectively. In the Andeans, 400 and 41 regions were in the top 5% and 1% PBS tails, respectively.

## Supplementary Material

Supplementary figures S1–S4 and tables S1–S6 are available at *Molecular Biology and Evolution* online (http://www.mbe.oxfordjournals.org/).

Supplementary Data
