# Supplementary material for: Shared Genetic Signals of Hypoxia Adaptation in Drosophila and in High-Altitude Human Populations
Source: Mol Biol Evol. 2015 Nov 17;33(2):501–17. doi: 10.1093/molbev/msv248 (PMC4866538; doi:10.1093/molbev/msv248)
Supplement: Supplementary Data [file supp_33_2_501__index.html]

Shared genetic signals of hypoxia adaptation in Drosophila and in high-altitude human populations. — Shared Genetic Signals of Hypoxia Adaptation in Drosophila and in High-Altitude Human Populations — Shared Genetic Signals of Hypoxia Adaptation in Drosophila and in High-Altitude Human Populations — Supplementary Data 

# Shared Genetic Signals of Hypoxia Adaptation in *Drosophila* and in High-Altitude Human Populations

## Supplementary Data

files

- Supplementary Data - pptx file
- Supplementary Data - xlsx file
