# Supplementary material for: Shared Genetic Signals of Hypoxia Adaptation in Drosophila and in High-Altitude Human Populations
Source: Mol Biol Evol. 2015 Nov 17;33(2):501–17. doi: 10.1093/molbev/msv248 (PMC4866538; doi:10.1093/molbev/msv248)
Supplement: Supplementary Data [file supp_msv248_Jha_MBE_SupFigures_Revision.pptx]

## Slide 1
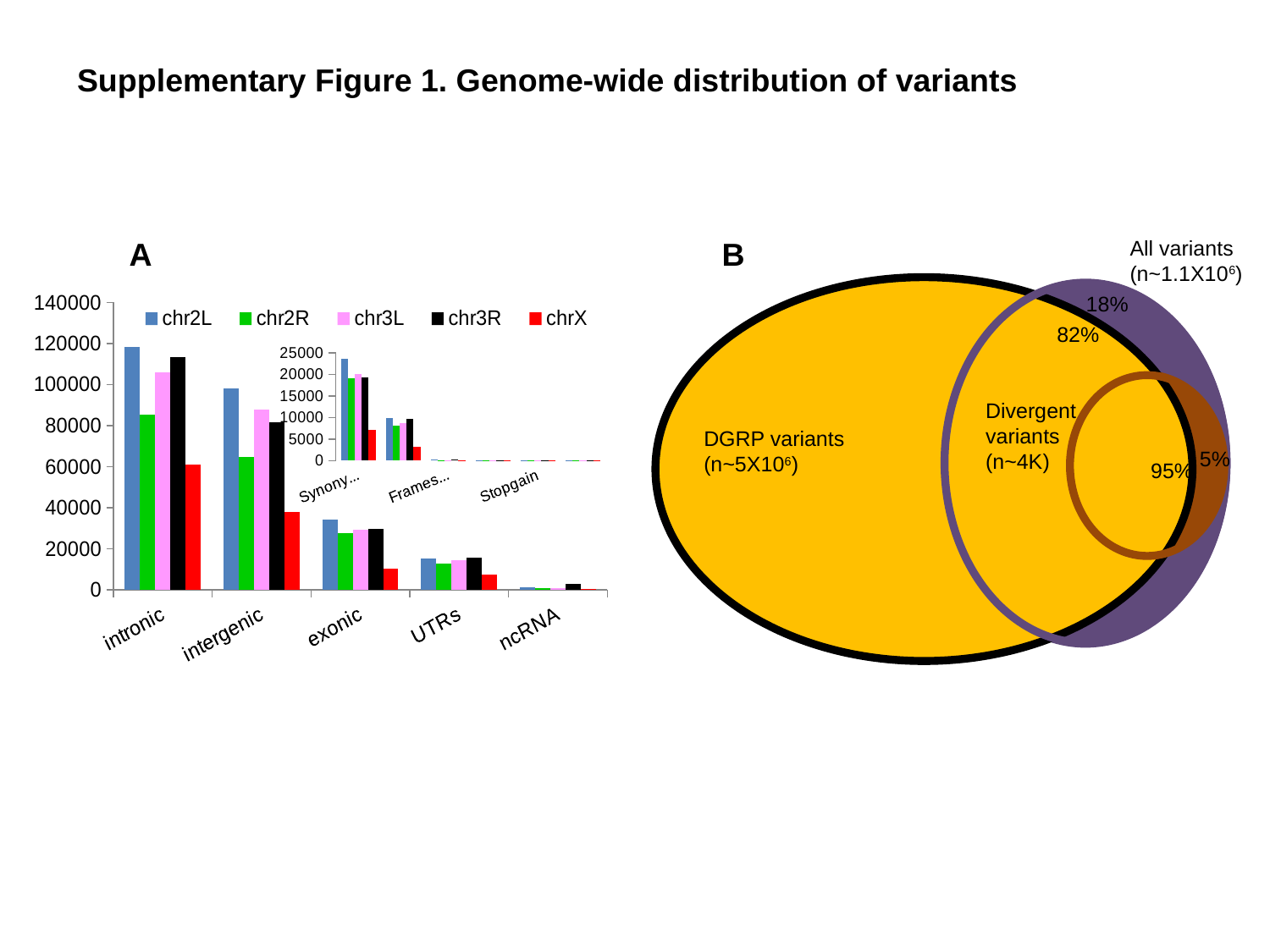

Supplementary Figure 1. Genome-wide distribution of variants
A
All variants
(n~1.1X106)
18%
82%
Divergent variants
(n~4K)
DGRP variants
(n~5X106)
5%
95%
B
### Chart
| Category | chr2L | chr2R | chr3L | chr3R | chrX |
|---|---|---|---|---|---|
| intronic | 118168.0 | 85438.0 | 106089.0 | 113428.0 | 60973.0 |
| intergenic | 98303.0 | 64811.0 | 87876.0 | 81550.0 | 38026.0 |
| exonic | 34090.0 | 27570.0 | 29204.0 | 29677.0 | 10466.0 |
| UTRs | 15355.0 | 12683.0 | 14522.0 | 15531.0 | 7357.0 |
| ncRNA | 1191.0 | 647.0 | 696.0 | 2704.0 | 351.0 |
### Chart
| Category | chr2L | chr2R | chr3L | chr3R | chrX |
|---|---|---|---|---|---|
| Synonymous | 23691.0 | 19149.0 | 20120.0 | 19378.0 | 7142.0 |
| Nonsynonymous | 9870.0 | 8013.0 | 8717.0 | 9669.0 | 3117.0 |
| Frameshift | 159.0 | 110.0 | 106.0 | 146.0 | 54.0 |
| Nonframeshift | 25.0 | 29.0 | 24.0 | 28.0 | 21.0 |
| Stopgain | 73.0 | 49.0 | 40.0 | 58.0 | 22.0 |
| Stoploss | 16.0 | 16.0 | 9.0 | 17.0 | 5.0 |

## Slide 2
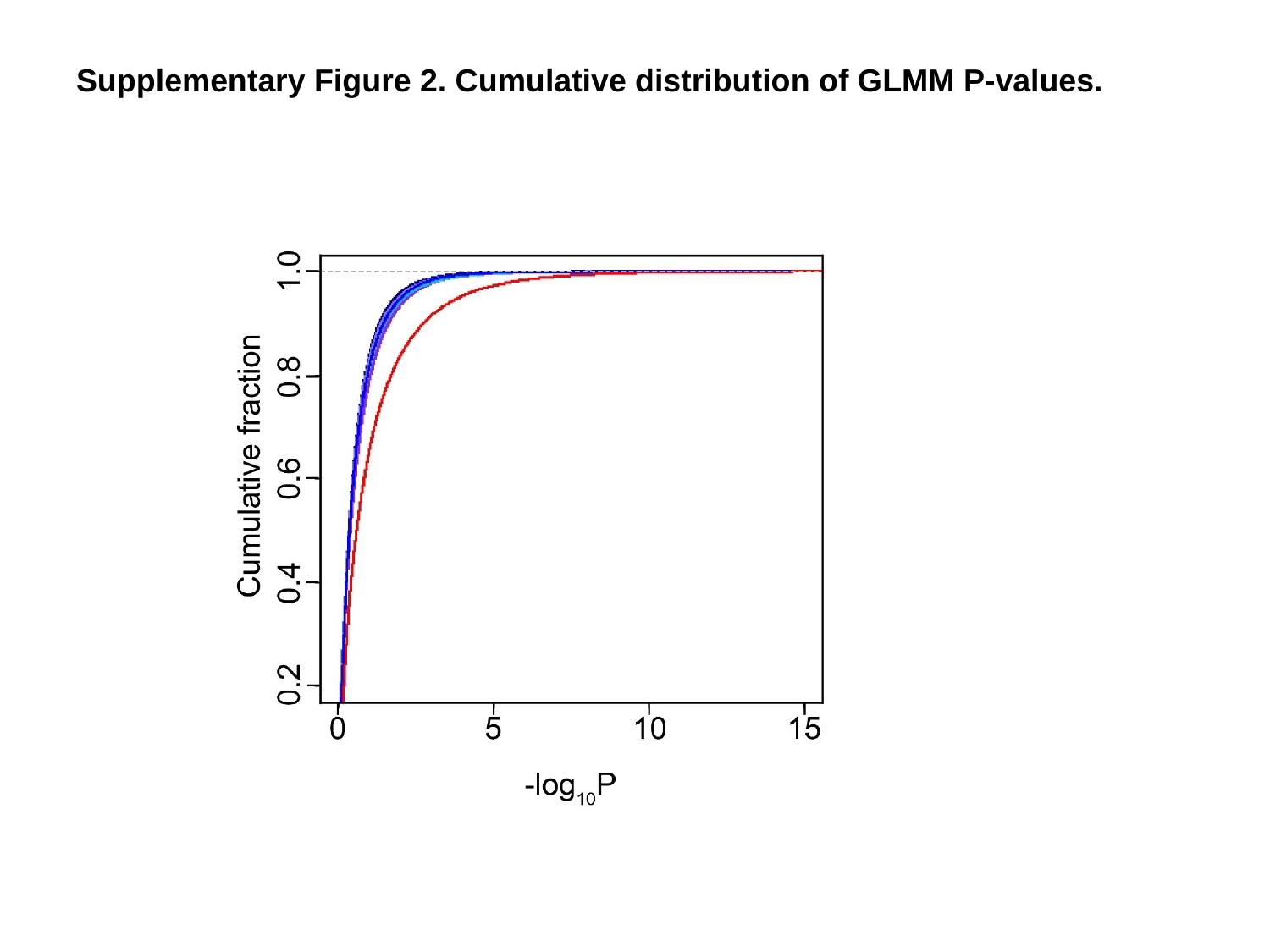

Supplementary Figure 2. Cumulative distribution of GLMM P-values.

## Slide 3
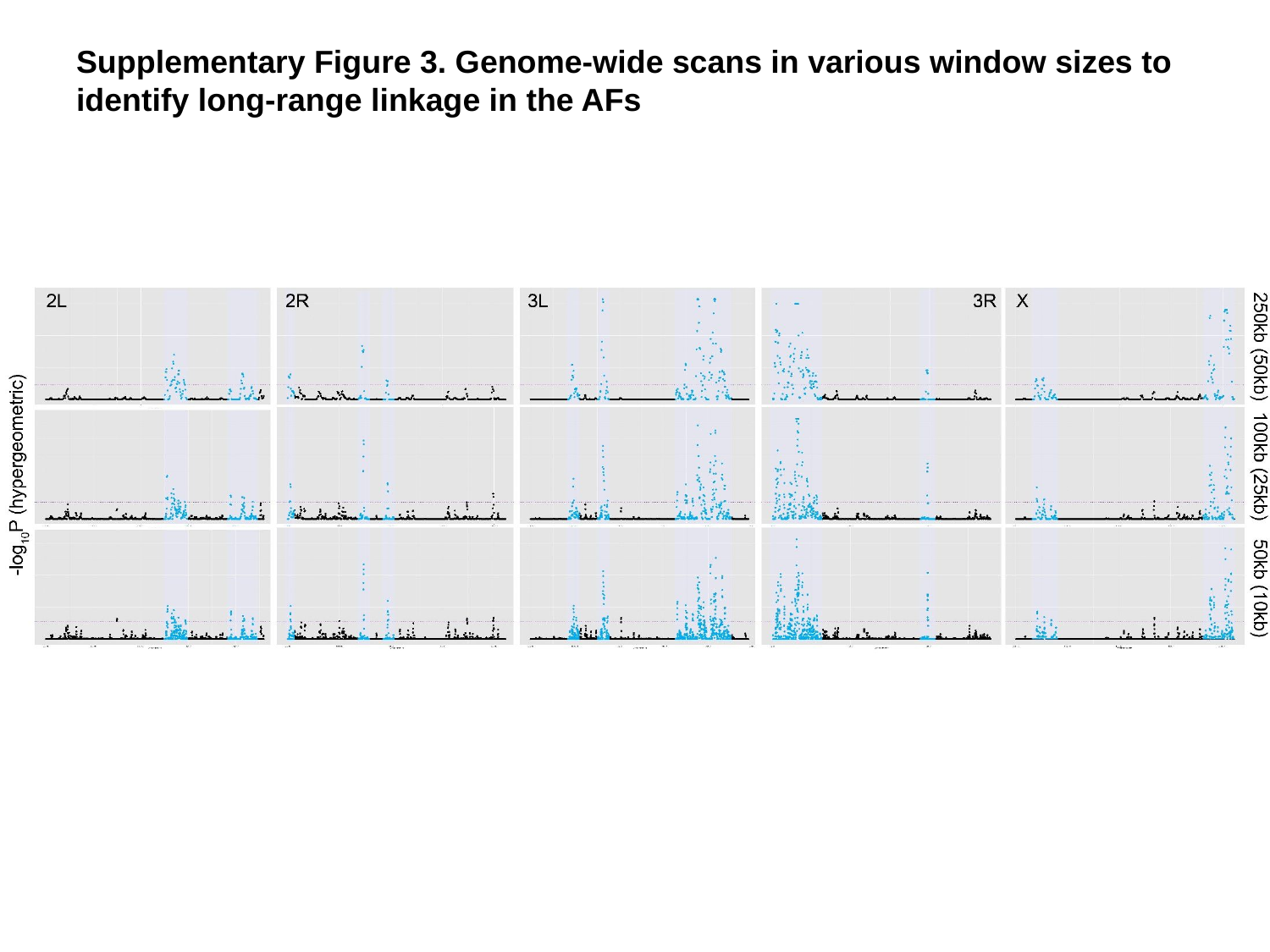

Supplementary Figure 3. Genome-wide scans in various window sizes to identify long-range linkage in the AFs

## Slide 4
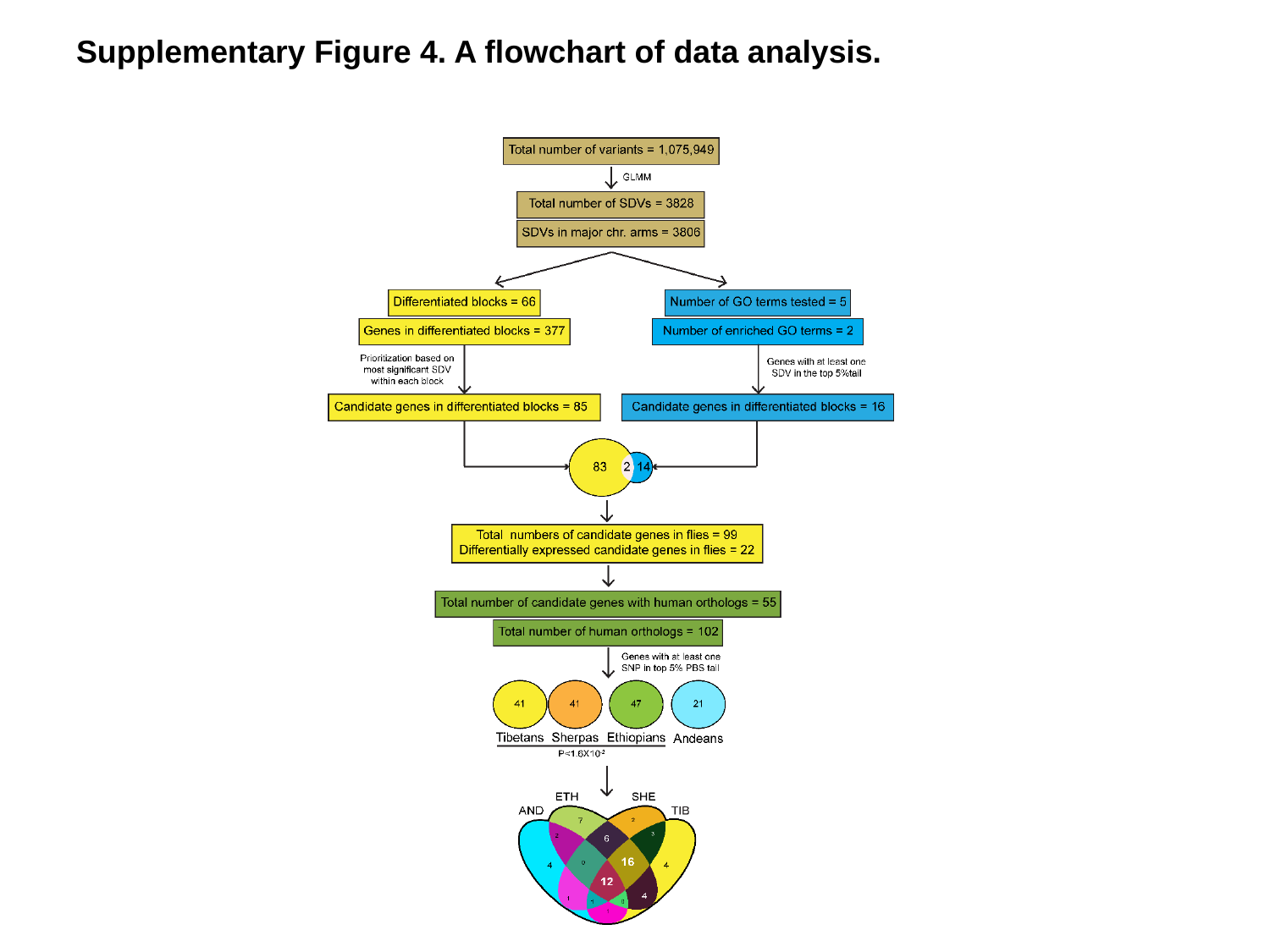

Supplementary Figure 4. A flowchart of data analysis.
